# The two-faced process of learning and the importance of Janus-faced solutions

**DOI:** 10.1038/s41539-023-00210-w

**Published:** 2023-12-14

**Authors:** Robin Samuelsson

**Affiliations:** Department of Scandinavian Languages, Uppsala University, Thunbergsv 3L, 751 20 Uppsala, Sweden

**Keywords:** Education, Human behaviour, Human behaviour, Education, Interdisciplinary studies

## Abstract

Significant developments have been made to our understanding of how children learn, putting essential pieces to the puzzle of what it means to be human. Theories of learning are, however, headed in diverging directions, and this perspective paper argues that this dispersion can recapitulate recurring schisms in developmental and learning sciences about learning as a predominantly individually constructed or socially transferred process. It is argued that this opposition is unnecessary and that an encompassing understanding of learning should consider both directions. This conciliatory approach considers how humans learn from others and what is known while exploring new solutions. This is important for understanding learning in childhood, seeing learning as a simultaneously individual and social process where humans actively explore and exploit knowledge about the world around them. Framing learning by the metaphor of a Janus face, looking back at what is known while exploring new knowledge, becomes illuminating for understanding learning and provides an essential background for designing educational practices based on active learning.

Human beings accomplish a remarkable feature of learning in childhood: the ability to gather information from interaction with rich social and physical environments and process this information into structured and actionable knowledge^1^. Understanding human learning is essential and, not least, intriguing because learning captures a vital facet of what it means to be human. However, if we look into the current state of learning theories, there is an increasing dispersion in our understanding, where theories point in seemingly different directions. This paper argues that the current state of learning theories poses an unnecessary risk of recapitulating some long-lasting debates about human learning as a predominantly individual or social process and proposes a conciliatory approach building on the metaphor of human learning as a Janus-face, looking back at the known and exploring the unknown. The paper shows how such a unified account of learning can be informative for learning sciences research and the design of educational environments and programmes.

A major line of divergence in education debates has long been the role of knowledge as individually constructed or mainly as a transmission process, socially traded between generations. This conforms to how classrooms and learning environments are constructed, for example, in what role the teacher takes, how the classroom is set up, and what tools are allowed in the process. Much of this debate mirrors the interpretations of Piaget and Vygotsky, where Piaget often stands for individually constructed knowledge and Vygotsky for the role of sociocultural scaffolding^2^. While significant disagreements exist on whether these characteristics withstand close reading^3^, these fundamental disagreements continue to fuel debates in education and inform research in the sciences of learning and development^4,5^. In Piaget’s conception of learning, the role of individual actions working to construct a causal understanding of the physical world was key^6^. Whereas for Vygotsky, the interaction with social interlocutors and external cultural tools were the main drivers in the learning and developmental processes^7^. Put bluntly, in constructivist learning, learning begins with the individual child as active in their learning. In sociocultural learning theories, learning begins from the sociocultural environment that is internalised in the learning process, often through educational scaffolding in the zone of proximal development.

Problems can, however, arise from a too-stark view of learning, that has both scientific and educational consequences. Much recent research falls into this dichotomous view of learning and risks regurgitating schisms that separately treat learning as an individual construction or a socially transmitted process. This is unfortunate and should be worked to be reconciled if we are to understand and use the full complexity of human learning. A unified account of learning must inevitably contain both these dimensions of learning. The following section reviews current literature that builds on these two faces of the learning sciences and then develops the Janus-faced alternative unifying these.

## The two faces of human learning

While the debates on constructivist vs. sociocultural learning mechanisms have, in many ways, been nuanced, traces of their underlying conceptions of learning continue to inform research. They can be traced in lines of enquiry in research efforts today and continue to affect the construction of learning environments^8^. Here, some recent evidence on social learning and constructivist mechanisms are reviewed to be later nuanced in light of recent evidence.

### The literature on social and cultural learning

One direction in the literature concerns how we learn from others, fundamentally children’s propensity for social learning, by how knowledge is transmitted between individuals interacting with or by learning from observation of what others do^9,10^. One form of social learning concerns cultural learning, being learning of culturally transmitted knowledge and understanding of cultural tools. This has been an area of fruitful research in the past decades showing how cultural learning is fundamentally undergirded by human cooperation, sociality, and builds on a shared understanding of goal-directed cultural behaviours^11^. Children imitate other cultural models with astonishing precision and fidelity^12^. Learning, in this sense, is fundamentally a social phenomenon and underscores how children cannot discover important information about the natural and cultural world just by themselves^8^.

In modern societies, learning has often been organised through a classroom model, where teachers lead the class through instruction as an efficient and effective way of transferring understanding to larger groups of children. A similar movement can be seen in educational psychology on the role of direct instruction from a teacher^13^. Often, this research has proved the effectiveness of techniques and tools for instructing children to learn what is being taught^9^. Based on this assumption, it follows how teaching children through direct instruction becomes a critical element of educational approaches.

### Exploration, play and constructivist learning

Another direction in learning theories emphasises active learning from the exploratory and constructionist side of human learning. Here, advancements show children’s ability to make probabilistic inferences from prior understanding and reason based on available information. Astonishingly, children solve the problem of induction^14^, and act in probabilistically rational ways based on the information gathered^15,16^. Crucially, humans are embodied and active agents that move and interact with the environment in ways that affect their understanding^17^, and children, from an early age, actively search for evidence about the world around them^18^.

An example is how children’s playful and explorative interaction with objects can be a way for children to actively construct a basic understanding of the things they play and tinker with^19^. This type of constructivist learning is very much adjacent to Piagetian constructivist learning. From this, children form hypotheses about the world that can be tried through experimentation in exploration and play, forming a basis for shaping children’s understanding of the world. This underpins how children construct a generalisable understanding from free play and exploratory play^20^. This research programme has sometimes provided a picture of children as small ‘scientists’^21^, capturing the active exploratory and playful sides of how children learn. Today, this type of learning is infused with various cultural tools, such as toys and technologies that children play with^22^, often educationally designed to shape how knowledge is constructed^23^.

### Beyond learning as either a social or individualistic process

At a surface level, these research programmes examine different learning experiences for children and go back to long-lasting educational questions that mirror the debates on Piagetian constructivism or Vygotskyan sociocultural learning, and the fundamental philosophical questions about learning—*do children construct knowledge*, or *is it taught to them?* These questions point to different views of human learning. The answer argued in this paper is that children importantly learn from social transfer and individual construction, which should be embraced in our learning models. Learning is about forming experience into actionable understanding. However, how one has answered these questions has had starkly different outcomes for scientific research and educational policies. This debate has had educational implications, where researchers and pedagogues have tended to camps favouring either an instructionist or constructionist view of learning, resulting in different educational environments^3^ that have relied on either heavily teacher-led pedagogies or child-directed educational alternatives.

Instead of cementing the dispersion, taking a conciliatory view, using the metaphor of a Janus-face that considers how learning centrally involves taking what is known and using this for future actions as the double-faced view for understanding human learning. We argue that going beyond the dichotomy between theories in the sciences of learning and embracing the double-sided nature of learning is informative for shaping a more encompassing theory of learning and creating better educational designs that effectively take advantage of the plurality of learning sciences in an integrated way.

## Janus-faced learning and educational alternatives

How can this theoretical incompatibility be reconciled? We propose that conceptualisations of learning should embrace both strands of the sciences of learning for a complete picture of real-life learning. Furthermore, humans’ unprecedented learning and cognitive abilities rely on our ability to read and further conceptualise the social, physical and biological world around us, store and transfer this knowledge, and create new possibilities from this understanding. The learning and knowledge-generating processes thus need both sides of learning where knowledge and skilful use of what we learn are key parts. This is a dynamically unfolding process, where learners may rely on instructed material socially attained from others at one stage while also constructing their conceptions and introducing innovation to understanding and action at different stages of a learning process.

Theories of learning should embrace that, as humans, we have to explore new information and use the information already available from the cumulated, stored and traded cultural knowledge around us. Under some conditions, learning from others in a cultural setting can be the most advantageous strategy. At other times, exploring novelty or playing with what is already known can be a useful way to decipher information for oneself, possibly discovering new possibilities^24^. Both these modes of engagement are equally central for humans. People infer understanding from trial-and-error and learn from others in structured environments. For example, a child might play with an object such as a swing, inferring an understanding of it and later revising this into a more scientific understanding in conversation with an older peer or adult at the playground^25^.

A core realisation is that exploratory and social learning modes have different virtues. Learning from others is an effective way of acquiring usable information and skills^14^ and a sine qua non to be part of a cultural environment^12^. Still, it also limits the potential for innovation^26^, as shown in how children stop searching for solutions when answers are given^24^. Thus, an exploratory and playful learning environment can spur children toward discovery learning, as an environment with limited ready-made information for children spurs children’s exploration^27^. Another example concerns that children who engage in imaginative play forms create new scenarios and goals from what they explored during previous learning processes^8^. In this manner, a playful approach can transform the child’s understanding and potentially even innovate their own or the cultural knowledge they play with^28^.

This double-sided nature of learning is a critical insight for education as it underscores the importance of accumulated knowledge and active use of it as part of the educational process. Here lies educational potential in a Janus-faced learning alternative, realising both the power in humans’ accumulated knowledge and the innovative potentials stemming from exploration and play-based approaches to learning. This is effectively used in several contemporary examples of active learning approaches^29^, where the critical educational question becomes how to create learning activities that build on tools and understandings of the past, for example, science^30^ or literature^31^, and using these in learning activities that foster children’s exploration and joy of learning. A conciliatory approach to learning can help us design learning environments conducive to human learning and the nature of childhood, while also informed by the big questions about learning and our current answers. Taking the metaphor of the Janus-face, looking back at what is socially and culturally known and using knowledge to explore the world and try new solutions is a fitting metaphor for human learning that embraces both strands of the sciences of learning.

If we take this unified view of how young children learn, we are given important pieces of understanding through how children choose exploratory approaches to problem-solving when less is known and draw on social learning from others when reasonable solutions are at hand^32^. A key component of human action and learning is knowing where to look—how to actively select among the abundant input sources of real-life situations^20^. Knowing where to explore is a key problem for exploratory learning theories^27^—and these decisions are informed by both exploratory experiences and/or social cues. Often, these modes of understanding are interfolded when successful learning occurs in highly creative learning processes where learners engage at the height of their abilities. For example, learning in guided play scenarios balances child-initiated curiosity and social scaffolding^2^ often in enriched learning environments. In this play-based educational setting, a teacher carefully designs learning environments with tools children are likely to explore and learn from and applies scaffolding in the learning process where appropriate. In this sense, a guided play programme provides a learning environment that intersects the discussion on learning as individual or social, stemming from the previously referenced debates on learning and teaching (such as learning in Piagetian constructivist vs. Vygotskyan sociocultural environments). It instead dynamically incorporates both forms of learning when appropriate in the learning process. This type of learning environment both takes advantage of children’s propensity to explore for new information and also for the power of the vast cumulated human cultural and scientific understanding. Another example is the pedagogical approaches of Montessori preschools and schools, promoting active, hands-on learning with select tools and scaffolding, which is a well-tested^33^ alternative to typical classroom approaches to teaching and learning at the more constructivist side. In yet another way, Tools-of-the-mind programmes^28^ use cultural tools as a major component in a play-based sociocultural alternative implementing a balance of play and teacher-scaffolded activity.

## Janus-faced learning embodied in real-life learning scenarios

The childhood period is naturally predisposed to exploration, and humans have core biases for learning—that change with new experiences, children’s active exploration, and social scaffolding^29^. In real-life learning, these modes intermix and people today are often required to be both good social learners and creative to thrive in knowledge professions. In a Janus-faced view, learning is a process *pendulating between exploration and exploitation, between individual and social learning mechanisms*. A core component thus becomes when choosing one mode of learning over another, and this is a key balancing act between knowledge and skills that is key to a well-rounded contemporary education that both need to engage active learners, while also drawing on the vast knowledge of the past. Not least, the call for a 21st-century education, such as the role of education as an arena for embodied learning that also incorporates elements of creative skills, making and imagination^37^, requires theories of learning that can both include transmission of the cumulated scientific and technological understanding while working to foster innovative education that goes beyond the mere transmission of information.

The Janus-faced nature of human learning means that we must take what we know from the past and generate usable knowledge for future actions. However, taking a culturally accepted solution limits the possibility of discovery, innovation, and better solutions and actions—this is at the core of the puzzle of human learning and risks being missed in too polarised views of learning being unleashed in pedagogical practices. Humans are embodied and active learning agents that actively search for new solutions and learn from others. Moving and trying new things out is always an option for humans during real-life active learning scenarios. This should be fundamentally incorporated when designing learning environments for children. Children’s education is a balancing act between playful, exploratory discovery and teaching of what is known—in the learning sciences literature, this is an area of exchange that has not been sufficiently uncovered.

The Janus-faced balancing act is at the heart of developing education conducive to human learning, reconciling accumulated knowledge with children’s joy of learning. Understanding this interplay is already at play in several successful pedagogical alternatives^28–31^ that have moved beyond stilted debates on instruction vs. construction of knowledge^3^, and powerfully incorporate both these poles in balanced learning environments that are knowledge-rich, materially and socially engaging. This balancing act of learning from what is already known and actively creating new sources of experience is vital for understanding human learning and recalibrates pedagogical discussions toward a fruitful agenda of creating rich educational experiences for children.
